# Tuberculose parotidienne: un diagnostic oublie, à propos de 2 cas et revue de littérature

**DOI:** 10.11604/pamj.2018.29.203.14682

**Published:** 2018-04-06

**Authors:** Mohammed Chouai, Azeddine Lachkar, Adel Abdenbi Tsen, Fahd El Ayoubi, Rachid Mohamed Ghailan

**Affiliations:** 1 Service d’ORL, et de Chirurgie Maxillo-Faciale, CHU Mohamed VI, Oujda, Maroc

**Keywords:** Parotide, tuberculose, parotidectomie

## Abstract

La tuberculose parotidienne reste une localisation très rare au niveau de la sphère ORL. Elle se présente sous forme d'un tableau clinique trompeur faisant confusion avec d'autres pathologies de la glande parotide, notamment la pathologie tumorale. De plus, sa méconnaissance par des praticiens, augmente le risque de passer à côté du diagnostic. Souvent, le diagnostic est une surprise histologique sur une pièce d'exérèse après une parotidectomie exploratrice. Cependant, son traitement est avant tout médical si le diagnostic positif est bien établi. Nous rapportons l'observation médicale de deux nouveaux cas âgés respectivement de 27 ans et 49 ans qui consultent dans notre formation pour des tuméfactions parotidiennes. Les examens radiologiques étaient en faveur de lésion kystique intraparotidienne. Les 2 patients ont bénéficié d'une exérèse dont l'étude anatomopathologique et immuno-histochimique était en faveur d'une tuberculose primaire de la parotide. L'évolution par la suite a été favorable sous traitement antituberculeux.

## Introduction

La tuberculose est une infection granulomateuse chronique due à Mycobacterium tuberculosis ou bovis qui peut toucher tous les organes. Constitue toujours un problème de santé publique dans notre contexte. La localisation pulmonaire demeure la principale atteinte, Au niveau de la sphère ORL, l'atteinte ganglionnaire est la plus fréquente. La localisation isolée de la tuberculose au niveau des glandes salivaires et notamment de la parotide, est extrêmement rare et ce, même dans les pays à forte endémie tuberculeuse comme le Maroc. Aucun élément clinique, radiologique et biologique ne permet d'orienter le diagnostic. Sa présentation peut mimer une tumeur maligne [1].

## Patient et observation

**Figure 1:**
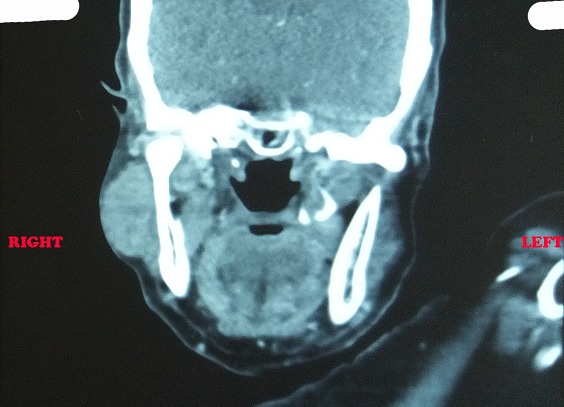
TDM en coupe coronale passant par la parotide

**Figure 2:**
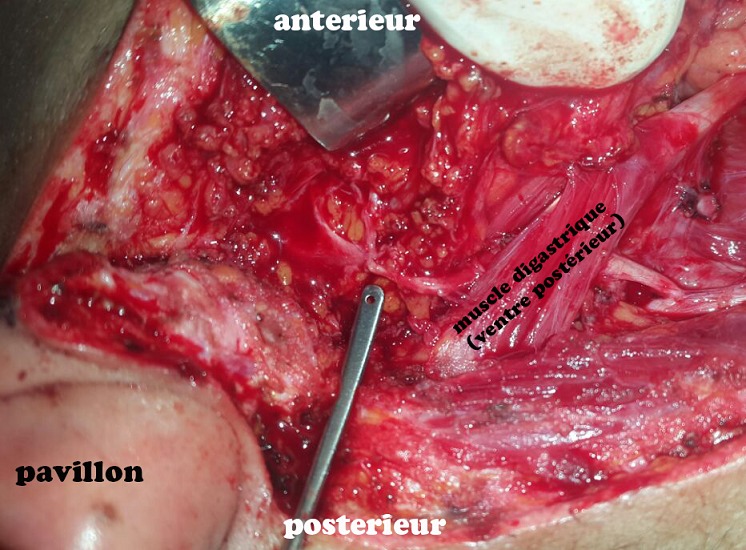
Parotidectomie exofaciale avec conservation du nerf facial

**Figure 3:**
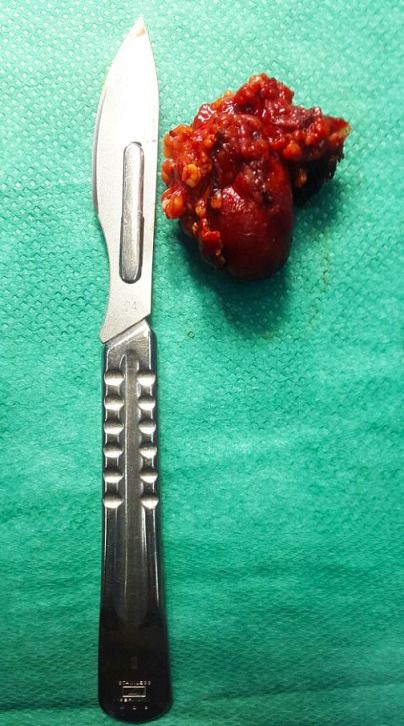
Pièce opératoire

**Observation 1:** il s'agissait d'une femme âgée de 27 ans, sans antécédents pathologiques particuliers, qui a présenté une tuméfaction isolée de la région parotidienne droite, évoluant depuis 14 mois. À l'examen clinique: La patiente était en bon état général, apyrétique. La tuméfaction était prétragienne et mesurait 4cm de grande axe, ferme, bien limitée, mobile et indolore, associes de multiples adénopathies cervicales homolatérales jugulo-carotidiennes dures et fixées. La parotide controlatérale était sans particularités. Par ailleurs la patiente ne présente pas de gêne à la mastication, ni trismus ou d'issue de pus en endobuccal, ni paralysie faciale périphérique et les voies aéro-digestives supérieures étaient libres. L´échographie cervicale a montré un processus de la glande parotide hypoéchogène hétérogène renfermant des zones de nécroses. Une tomodensitométrie cervico-faciale a montré une collection intraparotidienne droite, hétérogène, cernée par une coque périphérique irrégulière et rehaussée (Figure 1). A L´IRM, la lésion était au dépend du lobe superficiel de la parotide gauche, ovalaire de 11 x 11 x 13mm. Le signal était mixte avec une composante liquidienne centrale et charnue périphérique en hypo T2 prenant le contraste après Gado. En diffusion, le coefficient de diffusion était bas. Ses caractéristiques étaient suspectes de malignité. L'intradermoréaction à la tuberculine était positive, le reste du bilan biologique était normal. La radiographie pulmonaire ne montrait aucuns signes d'une possible localisation pulmonaire. Le diagnostic d'un cancer parotidien a été fortement suspecté. Pour avoir une preuve histologique, nous avons décidé de réaliser une parotidectomie exo-faciale partielle avec dissection et conservation du nerf facial (Figure 2). L'examen anatomopathologique de la pièce opératoire (Figure 3) a conclu à une tuberculose parotidienne primaire et ganglionnaire cervicale. Ceci a été confirmé par l'examen histologique définitif. La patiente a été mise sous traitement antibacillaire à type de schéma 2RHZ/4RH pendant six mois avec une évolution favorable avec un recul de 9 mois après la fin du traitement.

**Figure 4:**
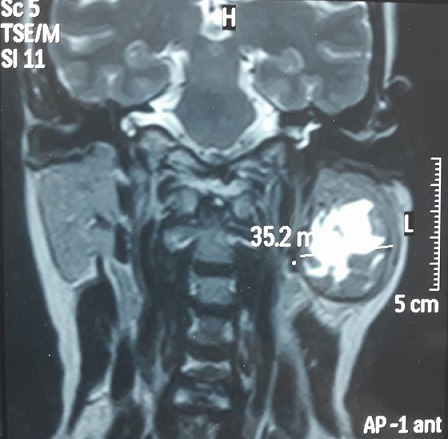
IRM en coupe coronale en pondération T2

**Figure 5:**
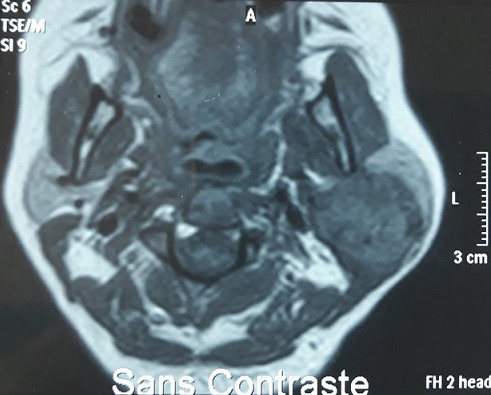
IRM en coupe axiale T1 sans injection de Gadolinium

**Figure 6:**
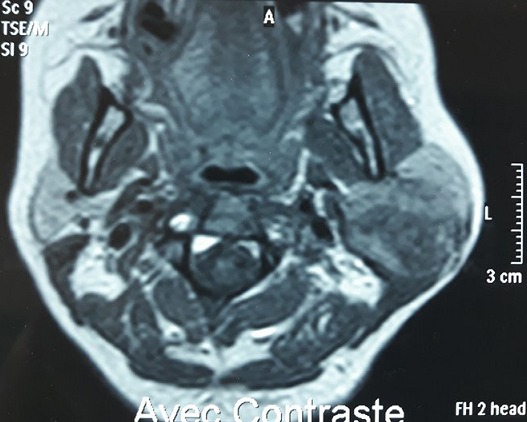
IRM en coupe axiale T1 après injection de Gadolinium

**Observation 2:** une femme de 49 ans, sans antécédent pathologique notable, a consulté dans notre formation pour une masse sous-angulo maxillaire gauche évoluant depuis environ 5 ans. Il n'existait pas de signes cliniques associés, notamment pas de douleurs, de fièvre, d'amaigrissement, de gêne à la mastication, de trismus ou d'issue de pus au niveau des orifices salivaires. La jeune femme était en bon état général et présentait une tuméfaction de la région parotidienne gauche, ferme, indolore, mesurant environ 5 cm de grand axe, fixe. Il n'existait pas de limitation de l'ouverture buccale, ni de paralysie faciale périphérique. L'examen de la cavité buccale et de l'orifice du canal de Sténon ne mettait pas en évidence d'inflammation ni d'issue de pus. L'examen des aires ganglionnaires cervicales permettait de palper quelques adénopathies infra-centimétriques homolatérales, jugulo-carotidiennes. Le reste de l'examen ORL et général était sans particularité. Une échographie cervicale a était faite à montrer une lésion tumorale faisant 35 x 34 x 35mm d'echostructure mixte à prédominance liquidienne centrale au dépend du pole inferieure de la parotide gauche. L'IRM parotidienne a objectivé une masse intra-parotidienne inferieure gauche mesurant 4 cm de grand axe, grossièrement arrondie, de contours réguliers, entourée d'une capsule en hyposignal T2, avec un centre en hypersignal T2 franc, hypersignal diffusion, avec un rapport ADC tumeur sur parenchyme sain supérieur à 2. Ceci était en faveur d'un adénome pléomorphe, néanmoins l'aspect hétérogène de la masse et la présence des adénopathies jugulo-carotidiennes homolatérales n'excluant pas une dégénérescence maligne. (Figure 4, Figure 5, Figure 6). Le reste du bilan biologique, ainsi que la radiographie pulmonaire était normal. Le malade fût opéré, bénéficiant d´une parotidectomie conservatrice du nerf facial. L'étude histopathologique de la paroi du kyste a retrouvé un granulome épithélio-gigantocellulaire avec de la nécrose caséeuse. La mise en culture du liquide de ponction sur milieu de Lowenstein-Jensen a mis en évidence un bacille acidoalcoolorésistant, ce qui nous permettait ainsi de confirmer le diagnostic de tuberculose primaire de la glande parotide. La jeune femme a reçu un traitement médical d'antituberculeux associe une phase initiale de 2 mois à base de rifampicine, isoniazide, ethambutol, pyrazinamide, suivie d'une phase de consolidation de 4 mois associant la Rifampicine et l'Isoniazide. L'évolution a été favorable avec un recul de 6 mois après la fin du traitement.

## Discussion

La tuberculose parotidienne est une entité rare de la tuberculose extrapulmonaire et particulièrement de l'atteinte ORL extraganglionnaire. Après que Robert Koch à découvert le bacille responsable en 1882, le premier cas de tuberculose parotidienne secondaire a été rapporté en 1894 par De Paoli, Von Stubenrauch a décrit un deuxième cas en 1893, qui est d'atteinte primaire. Depuis ce temps à nos jours la littérature a rapporté 220 cas. C'est une pathologie qui peut atteindre tous les âges. Mais souvent, l'adulte jeune entre 20 et 40 ans, reste plus incriminé. L'enfant est rarement touché. Les deux sexes, féminin et masculin, sont touchés de façon équivalente. Quatre-vingt-dix pourcent des cas publiés proviennent des continents Africain et asiatique, des pays où la tuberculose constitue un problème de santé publique. La localisation parotidienne pose un véritable défis de diagnostic différentiel pour les praticiens, du fait du tableau clinique non spécifique et que plusieurs examens paracliniques sont non concluants [1]. Tous les facteurs qui favorisent la survenue de la tuberculose dans toutes ses formes, interviennent aussi dans l'atteinte parotidienne, à savoir le bas niveau socioéconomique, la promiscuité, le contage tuberculeux et les états d'immunodépression comme le VIH, le diabète et la malnutrition. Un état dentaire délabrant avec caries dentaires est suggéré avoir un rôle qui favorise l'infection. La consommation du lait cru non pasteurisé semble aussi être une cause puisque c'est une source de contamination par le bacille M. Bovis. La présentation clinique commune est une augmentation progressive de la taille de la glande sur une période de 2 à 6 mois en moyenne, indolore. Néanmoins, la durée d'évolution est variable et peut atteindre plusieurs années. Cette tuméfaction prend un aspect ferme et dur, parfois nodulaire, avec des degrés variables de fixation par rapports aux plans superficiel et profond, donnant un aspect pseudo tumoral. L'atteinte est souvent unilatérale, rarement qu'elle survient dans les deux glandes parotides à la fois [2]. Une fistulisation de la tumefaction peut orienter le médecin vers la pathologie tuberculeuse, mais généralement le plan cutané reste intact avec des légers signes inflammatoire en regard. Le trismus est rare dans les sialadénites tuberculeuses. Sa présence notamment avec une paralysie faciale périphérique fait douter une tumeur maligne de la glande parotide.

La tuméfaction parotidienne peut survenir de façon isolée comme elle peut être associée à des adénopathies cervicales peut orienter le praticien vers une pathologie tumorale maligne dans un tableau d'extension locorégional d'une tumeur parotidienne. Dans l'atteinte primaire, les signes d'imprégnation tuberculeuse à type de sueurs nocturnes, amaigrissement, asthénie et fièvre sont rarement présents [3]. L'échographie cervico-parotidienne est simple à réaliser, la raison pour laquelle elle est réalisée en première intention devant un tableau de tuméfaction parotidienne. L'aspect général est caractérisé par l'augmentation de la taille de la glande, avec une ou plusieurs lésions hypoéchogènes de taille variable, à limite nettes, sans renforcement postérieur. Les abcès froids tuberculeux parotidiens se présentent comme des collections hypoéchogènes ou anéchogènes mal limitées, avec renforcement postérieur, contenant parfois des échos intenses qui correspondent à des débris. Or, ces aspects peuvent se voir dans d'autres pathologies notamment l'adénome pléomorphe, les carcinomes, les lymphomes, les infections fongiques et parasitaires, les abcès à pyogènes ou les adénopathies intraparotidiennes [4]. La TDM cervico-faciale permet d'explorer la glande parotide dans toutes ses dimensions avec une grande sensibilité par rapport à l'échographie. Les coupes tomodensitométriques sont réalisées avant et après injection d'un produit de contraste iodé, d'une épaisseur de 5mm. Cependant, les aspects radiologiques trouvés ne sont pas spécifiques et ne permettent pas de corréler les lésions à la tuberculose parotidienne. En comparant nos 2 cas et les résultats de la littérature, il semble que l'aspect le plus fréquent est la présence d'une lésions à paroi épaisse, prenant fortement le contraste avec de la nécrose au centre, qui est pathognomonique de la tuberculose. Néanmoins des études récents ont concluent que tous les aspects peuvent être vus: rehaussement homogène de la glande parotide; rehaussement homogène avec un microkyste excentrique non rehaussé; lésion hypodense à paroi épaisse qui prend le contraste; lésions nodulaires hypodenses isolées ou confluentes. Or, la TDM reste toujours limitée [5]. L'IRM cervico-faciale permet de bien analyser la loge parotidienne et d'affirmer la nature parotidienne de la lésion. Elle est considérée comme supérieure à la TDM dans la détection et l'analyse d'un processus tumoral parotidien. A l'état normal, le parenchyme parotidien montre un signal T1 plus intense que le muscle mais moins que la graisse périphérique. La capsule glandulaire parait fine et régulière. Dans la tuberculose parotidienne, la lésion prend un hyposignal en T1 et un hypersignal en T2, qui peut être délimitée par une coque prenant le contraste après injection du Gadolinium. Cependant, cet aspect n'est pas spécifique. D'autres tumeurs bénignes très fréquentes de la parotide posent un problème de diagnostic différentiel, tel que l'adénome pléomorphe qui se manifeste par un hyposignal en T1 et un hypersignal en T2, prenant toujours le contraste mais parfois de façon hétérogène [5, 6].

Une radiographie pulmonaire doit être demandée de façon systématique à la recherche d'un éventuel foyer primitif. L'examen anatomopathologique est le gold standard dans le diagnostic de la tuberculose parotidienne. Les prélèvements pour cette étude proviennent de la cytoponction, d'un curetage biopsique de l'orifice fistuleux, d'une biopsie ouverte ou d'une pièce opératoire après parotidectomie. L'aspect typique est la présence de granulomes épithéloïdes giganto-cellulaire avec nécrose caséeuse qui est spécifique à la maladie et la distingue des autres granulomatoses, comme la sarcoïdose, où elle est absente [7]. Le diagnostic différentiel de la tuberculose parotidienne se fait surtout avec la pathologie tumorale, d'autres parotidites infectieuses, des maladies systémiques et les lithiases. Auparavant, la parotidectomie suivie d'un traitement antibacillaire constituaient le traitement de choix. Des auteurs attribuaient à la parotidectomie un rôle crucial pour la bonne diffusion des médicaments antituberculeux. Actuellement, tous les auteurs s'accordent sur l'inutilité de la parotidectomie et l'efficacité du traitement médical seul. D'après nos deux cas, nous avons conclu que les examens radiologiques, à savoir l'échographie, la TDM et l'IRM et biologiques ne confèrent pas des aspects spécifiques et ne permettent pas de poser le diagnostic d'où notre recours à une parotidectomie exofaciale a visée diagnostique et thérapeutique [8]. Le traitement antituberculeux au Maroc, s'intègre dans le cadre de la lutte antituberculeuse, préconisée par le ministère de la Santé Publique. Il s'agit d'un traitement standardisé gratuit, contrôlée et s'administre essentiellement en ambulatoire dans les centres de santé publique, l'hospitalisation est l'apanage des formes compliquées ou graves. Les formes cliniques de la tuberculose sont classées en quatre catégories selon les priorités thérapeutiques du programme National de la lutte Antituberculeuse; la catégorie I comprend les nouveau cas de TPM+ (tuberculose pulmonaire à microscopie positive) et les formes graves. La catégorie II associe les échecs thérapeutiques et les rechutes. La catégorie III comporte la TPM0 (tuberculose pulmonaire à microscopie négative), la TPM0C+ (tuberculose pulmonaire à microscopie négative et culture Positive), la PI (primo-infection) et la TEP (Tuberculose extrapulmonaire). Quant à la catégorie IV, elle englobe les cas chroniques et multirésistants. La tuberculose parotidienne tombe dans la catégorie III, et de ce fait le régime adopté est 2RHEZ/4RH. Il associe une phase initiale de 2 mois à base de 4 molécules antibacillaires dits de 1ère intention(RHEZ): rifampicine; isoniazide; ethambutol, pyrazinamide; suivie d'une phase de consolidation de 4 mois associant (RH) la Rifampicine et l'Isoniazide [8, 9]. Une supervision directe de la prise des médicaments se fait durant la phase initiale du traitement avec une surveillance bioclinique et/ou radiologique de l'efficacité et de la tolérance aux antibacillaires le long de la durée d'observance thérapeutique. Le risque de rechute ou de non-guérison malgré un traitement bien conduit est de 1%. Ces échecs sont dus à l'apparition de souches résistantes du BK aux antibacillaires [9].

## Conclusion

La tuberculose de la glande parotide est rare et peut se présenter sous différentes formes cliniques. prête souvent confusion avec la pathologie néoplasique. Du fait que le Maroc est un pays où la tuberculose est un problème majeur de santé public, le chirurgien ORL doit évoquer ce diagnostic devant toute tuméfaction parotidienne et ceci nécessite une connaissance des aspects épidémiologiques, cliniques et paracliniques permettant un diagnostic positif précoce avant d'arriver à la chirurgie vu que le traitement antibacillaire seul est efficace permettant la régression de la tuméfaction parotidienne et l'élimination du foyer tuberculeux; le recours à la parotidectomie à des visées diagnostique et/ou thérapeutique n'est plus d'actualité.

## Conflits d’intérêts

Les auteurs ne déclarent aucun conflit d'intérêts.
